# Measures of Patient Dissatisfaction With Health Care in Polycystic Ovary Syndrome: Retrospective Analysis

**DOI:** 10.2196/16541

**Published:** 2020-04-21

**Authors:** Luis R Hoyos, Manesha Putra, Abigail A Armstrong, Connie Y Cheng, Carrie K Riestenberg, Tery A Schooler, Daniel A Dumesic

**Affiliations:** 1 Department of Obstetrics and Gynecology University of California Los Angeles, CA United States; 2 Division of Maternal-Fetal Medicine Department of Obstetrics and Gynecology MetroHealth Medical Center, University Hospital Cleveland Medical Center Cleveland, OH United States

**Keywords:** PCOS, fibroid, Google, healthcare quality, infoveillance, infodemiology, medical education, health care, internet, satisfaction

## Abstract

**Background:**

Polycystic ovary syndrome (PCOS) is a common reproductive and metabolic disorder in women; however, many clinicians may not be well versed in scientific advances that aid understanding of the associated reproductive, metabolic, and psychological abnormalities. Women with PCOS are dissatisfied with health care providers, the diagnostic process, and the initial treatment of PCOS and seek information through alternative sources. This has affected the patient-physician relationship by allowing medical information acquired through the internet, whether correct or not, to become accessible to patients and reshape their health care perspective. Patient dissatisfaction with health care providers regarding PCOS raises questions about the responsibilities of academic institutions to adequately train and maintain the competence of clinicians and government agencies to sufficiently support scientific investigation in this field.

**Objective:**

The primary aim was to examine internet searching behaviors of the public regarding PCOS vs another highly prevalent gynecologic disorder. The secondary aim was to explore satisfaction with health care among patients with PCOS and their internet use. The tertiary aim was to examine medical education in reproductive endocrinology and infertility (REI) during obstetrics and gynecology (Ob/Gyn) residency as a proxy for physician knowledge in this field.

**Methods:**

Google search trends and StoryBase quantified monthly Google absolute search volumes for search terms related to PCOS and fibroids (January 2004 to December 2017; United States). The reproductive disorder, fibroids, was selected as a comparison group because of its high prevalence among women. Between female groups, monthly absolute search volumes and their trends were compared. A Web-based questionnaire (June 2015 to March 2018) explored health care experiences and the internet use of women with PCOS. REI rotation information during Ob/Gyn residency in the United States was obtained from the Association of Professors of Gynecology and Obstetrics website.

**Results:**

For PCOS (*R*=0.89; *P*<.01), but not fibroids (*R*=0.09; *P*=.25), monthly absolute search volumes increased significantly. PCOS-related monthly absolute search volumes (mean 384,423 searches, SD 88,756) were significantly greater than fibroid-related monthly absolute search volumes (mean 348,502 searches, SD 37,317; *P*<.05). PCOS was diagnosed by an Ob/Gyn in 60.9% (462/759) of patients, and 57.3% (435/759) of patients were dissatisfied with overall care. Among patients with PCOS, 98.2% (716/729) searched for PCOS on the Web but only 18.8% (143/729) of patients joined an online PCOS support group or forum. On average, Ob/Gyn residencies dedicated only 4% (2/43) of total block time to REI, whereas 5.5% (11/200) of such residencies did not offer any REI rotations.

**Conclusions:**

Over time, PCOS has been increasingly searched on the Web compared with another highly prevalent gynecologic disorder. Patients with PCOS are dissatisfied with their health care providers, who would benefit from an improved understanding of PCOS during Ob/Gyn residency training.

## Introduction

### Background

Polycystic ovary syndrome (PCOS) is a common reproductive and metabolic disorder in women, characterized by hyperandrogenism, menstrual irregularity, and polycystic ovarian morphology. Various PCOS phenotypes and different diagnostic criteria, as proposed by the National Institutes of Health (NIH), Rotterdam criteria, and the Androgen Excess and PCOS Society, often confuse both clinicians and the general public alike [1]. Moreover, many clinicians are not well versed in scientific advances that aid understanding of the reproductive, metabolic, and psychological abnormalities associated with PCOS. In a recent survey of gynecologists and reproductive endocrinologists, more than one-fourth of respondents did not know which PCOS diagnostic criteria they used and were unlikely to recognize associated comorbidities, complications, and benefits of lifestyle modification [2]. Worldwide, more than one-third of women see 3 different health care providers over 2 years before receiving a PCOS diagnosis [3]. Consequently, women with PCOS are dissatisfied with health care providers, the diagnostic process, and the initial treatment of PCOS [3-5] and seek information regarding weight loss, irregular menses, infertility, and excess hair growth through alternative sources [5-8]. This has led to the organization of educational events, such as the PCOS Awareness Symposium by PCOS Challenge, Inc, held at the University of California, Los Angeles (UCLA), in 2015, to provide the general public with up-to-date education on the diagnosis and management of PCOS as a crucial public health priority [9].

Public dissatisfaction with health care providers regarding PCOS care also raises questions about the responsibility of academic institutions to adequately train and maintain competence of clinicians in the care of women with PCOS. This responsibility also extends to government agencies to sufficiently support scientific investigation for research in PCOS.

Worldwide internet use now provides important health care information to everyone, with search engines, such as Google, improving the way one interacts with the world to ask questions and receive answers. Consequently, acquiring medical information through the internet has affected the patient-physician relationship by allowing information, whether correct or not, to become accessible to patients and reshape the perspective of their health care. In support of this, a study of online search behavior in the United States has shown that 72% of internet users use a search engine to obtain information about health care and clinical research [10-15]. Therefore, we hypothesized that increased public dissatisfaction with PCOS health care can be measured through exaggerated internet use.

### Objectives

The primary aim of this study was to examine internet searching behaviors by the public regarding PCOS vs another highly prevalent gynecologic disorder (ie, fibroids). The secondary aim was to explore satisfaction with health care and use of the internet in patients with PCOS using a survey. The tertiary aim was to examine medical education in reproductive endocrinology and infertility (REI) during obstetrics and gynecology (Ob/Gyn) residency as a proxy for physician knowledge in this field.

## Methods

### Patient Survey

Institutional review board approval was obtained from UCLA to develop an open online survey oriented toward women with PCOS. This questionnaire assessed health care satisfaction and whether respondents sought Web-based information related to their health care. The survey was developed after the PCOS Awareness Symposium held at UCLA in 2015 and started recruitment during June of that year until March 2018, following the identification of critical education and health care gaps. Recruitment was voluntary and accomplished through in-person requests, campus flyers, and advertisement on the Ob/Gyn departmental PCOS website with the wording “Do you have PCOS and are 18 years of age or older? If so, we would like to invite you to participate in a survey” [16]. The website was secured, contained relevant information for those interested in PCOS, and provided our survey to any patient with PCOS.

The usability and technical functionality of the survey were tested beforehand, and patients were informed of the survey’s purpose, length, and principal investigator. The survey’s length was 5 pages with six to eight questions per page, which were not adaptive, randomized, or with a review step. No incentives were offered to complete the survey, and no personal identifying information was collected. Women had the option to email or mail the survey to the research team. The data were then checked for completeness and entered by the research team into the secured UCLA research electronic data capture system. Paper copies were stored in locked offices, and data spreadsheets were kept on password-protected computers. Each completed item in the survey was analyzed independently, and missing response rates were provided.

### Google Trends

Google Trends is commonly used to monitor internet activities related to certain keywords by reporting an *index* of search activity. The fraction of queries that include the search term in a specific geography (ie, the United States) at a particular time relative to the total number of queries is measured by this index. The resulting numbers, defined as relative search volumes, are then scaled with the maximum value set at 100 [11,17]. Data regarding PCOS were compared with that of fibroids as another highly prevalent female reproductive disorder (NIH-defined PCOS, approximately 7% of reproductive-aged women; fibroids, approximately 70%-80% of women by age 50 years) [18,19]. Google Trends first examined the online search trend by generating monthly relative search volumes for the search terms *Polycystic Ovary Syndrome*, *PCOS*, or *Polycystic Ovarian Syndrome* from January 2004 to December 2017 in the United States. This trend for PCOS was then compared with that for fibroids with the search terms *Fibroid*, *Fibroids*, *Uterine Fibroid*, *Uterine Fibroids*, *Leiomyoma*, and *Myoma* over the same time interval.

Unlike Google Trends, search engine optimization (SEO) tool StoryBase (SEO.dk, Lyngby, Denmark) shows absolute search volumes but only does so for the preceding 12 months. Therefore, using this tool, the monthly absolute search volumes throughout 2017 were generated to calculate the yearly absolute search volume. The 2017 relative search volume was then obtained by adding the corresponding monthly relative search volumes obtained by querying the search terms from 2004 to 2017 with Google Trends. The absolute search volume from 2017 was then divided by the 2017 relative search volume, and from this, the absolute search volume for each relative search volume unit was calculated. Using this value along with the monthly relative search volume for each individual month in the study period, the corresponding monthly absolute search volumes were calculated.

Data were analyzed using SPSS version 22 (IBM Corp, Armonk, NY). Linear regression was used to examine the search trend of PCOS- and fibroid-related terms over time. The mean cumulative monthly absolute search volume of PCOS-related terms was compared with that of fibroid-related terms with an unpaired Student *t* test.

SEO tool StoryBase (SEO.dk, Google, Mountain View, California) was also used to obtain the top 200 questions related to the PCOS search terms. These questions were then classified according to their content (definition, treatment, fertility, etc), and a top 10 list of related questions was created.

### Obstetrics and Gynecology Residency Training

The percentage of block time devoted to REI during Ob/Gyn residency was examined, as information regarding PCOS-related questions correctly answered through the Council on Resident Education in Obstetrics and Gynecology (CREOG) examination was unavailable. The list of Ob/Gyn residency programs in the Association of Professors of Gynecology and Obstetrics (APGO) website was searched. Only programs in the United States were included, and all residency programs were classified as either university-based programs or non–university-based programs if they were either university-affiliated or community-based programs, respectively. For the latter, the Fellowship and Residency Electronic Interactive Database of the American Medical Association website [20] was reviewed. The characteristics of each program were searched, including numbers of graduating residents and REI rotations as well as percentages of rotation blocks spent in REI during residency. Rotation blocks instead of months were used as an outcome variable, as rotation duration was not consistently described. If listed, REI was assumed to be one block unless described otherwise. When one block was divided among multiple rotations, equal duration distribution was assumed. For example, if a block was labeled *REI, breast and US*, REI was recorded as one-third of a block.

## Results

### Patient Survey

Demographic characteristics of the 759 respondents to the UCLA-based PCOS survey are shown in Table 1.

Most respondents were diagnosed by an Ob/Gyn physician (462/759, 60.9%) and were often dissatisfied with explanations for the cause of their PCOS (319/759, 42.0%), treatment of their symptoms (389/759, 51.2%), and/or overall care of their symptoms (435/759, 57.3%; Table 2).

When asked about whether they had searched the internet for information about PCOS, 98.2% (716/729) of respondents replied yes, and 1.8% (13/729) replied no. Moreover, 3.9% (30/759) of women did not provide an answer. Only 18.8% (143/759) of respondents, however, had joined an online PCOS support group or forum.

**Table 1:** Demographic characteristics of survey respondents (n=759).

Demographic characteristics			Women, n (%)
**Age at survey response (years)**			
	18-25	366 (48.2)	
	26-35	283 (37.3)	
	36-50	89 (11.7)	
	≥51	7 (0.9)	
	Missing answers	14 (1.8)	
**Age o** **f first symptoms (years)**			
	<10	23 (3.0)	
	10-15	226 (29.8)	
	16-25	385 (50.7)	
	26-35	96 (12.7)	
	≥36	16 (2.1)	
	Missing answers	13 (1.7)	
**Race**			
	White	363 (47.8)	
	African American	107 (14.1)	
	East Asian	25 (3.3)	
	South Asian	70 (9.2)	
	Native American or Alaskan native	14 (1.8)	
	Native Hawaiian or Pacific Islander	1 (0.1)	
	Mixed	52 (6.9)	
	Other	117 (15.4)	
	Missing answers	10 (1.3)	
**Ethnic background**			
	Hispanic or Latino	103 (13.6)	
	Non-Hispanic or Latino	602 (79.3)	
	Missing answers	54 (7.1)	

**Table 2:** Survey of polycystic ovary syndrome patient satisfaction (n=759).

PCOS^a^ survey questions		Women, n (%)
**Have you received a formal diagnosis of PCOS by a health care professional?**		
	Yes	640 (84.3)
	No	59 (7.8)
	Not sure	50 (6.6)
	Missing answers	10 (1.3)
**What was the specialty of the doctor who diagnosed you with PCOS?**		
	Obstetrics and gynecology	462 (60.9)
	Family medicine	106 (14.0)
	Medical endocrinology	47 (6.2)
	Reproductive endocrinology and infertility	34 (4.5)
	Internal medicine	17 (2.2)
	Pediatrics	15 (2.0)
	Pediatric endocrinology	9 (1.2)
	Other specialties	42 (5.5)
	Missing answers	27 (3.5)
**How many doctors did you see for your symptoms before you received a diagnosis of PCOS?**		
	1	275 (36.2)
	2	213 (28.1)
	3	126 (16.6)
	4	58 (7.6)
	5	23 (3.0)
	≥6	35 (4.6)
	Missing answers	29 (3.8)
**At the time of your diagnosis, how satisfied were you with the explanation you received about the cause of PCOS?**		
	Completely satisfied	79 (10.4)
	Mostly satisfied	158 (20.8)
	Satisfied	179 (23.6)
	Not satisfied	319 (42.0)
	Missing answers	24 (3.2)
**At the time of your diagnosis, how satisfied were you with the initial explanation of your treatment options for managing your PCOS symptoms?**		
	Completely satisfied	56 (7.4)
	Mostly satisfied	119 (15.7)
	Satisfied	167 (22.0)
	Not satisfied	389 (51.2)
	Missing answers	28 (3.7)
**At the present time, how satisfied are you with the medical care you are receiving for your PCOS?**		
	Completely satisfied	38 (5.0)
	Mostly satisfied	106 (14.0)
	Satisfied	150 (19.8)
	Not satisfied	435 (57.3)
	Missing answers	30 (3.9)
**Since your diagnosis, have you sought medical care for PCOS from a health care provider other than the one who diagnosed your PCOS?**		
	Yes	322 (42.4)
	No	402 (53.0)
	Missing answers	35 (4.6)

^a^PCOS: polycystic ovary syndrome.

### Google Trends

**Figure 1:**
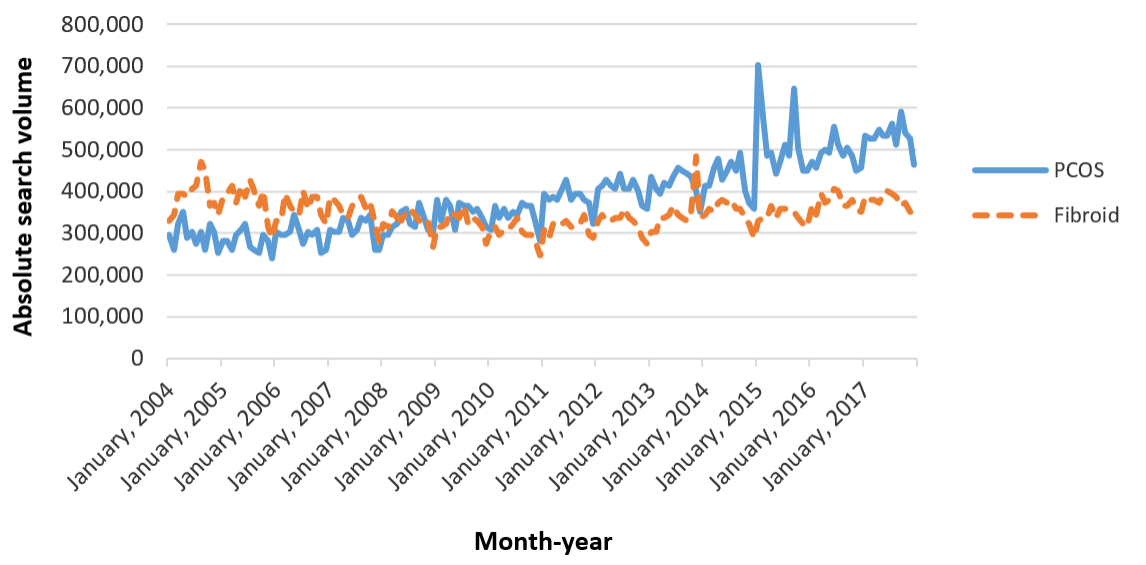
Monthly absolute search volumes for polycystic ovary syndrome and fibroids. PCOS: polycystic ovary syndrome.

Using Google Trends and SEO tool StoryBase (SEO.dk), we found that during the entire study period**,** there was a significant increase in the monthly absolute search volume of PCOS-related (*R*=0.89; *P*<.01), but not fibroid-related (*R*=0.09; *P*=.25), terms (Figure 1). Consequently, the mean monthly absolute search volume of PCOS-related terms between 2004 and 2017 was significantly greater than that of fibroid-related terms (PCOS: mean 384,423 searches, SD 88,756; fibroids: mean 348,502 searches, SD 37,317; *P*<.01) over the same time interval.

The SEO tool StoryBase (SEO.dk) also examined the top 200 questions related to PCOS search terms by popularity, and these questions were then classified according to content with the top 10 PCOS-related questions listed in Textbox 1. Ten different topics related to PCOS were included within the first 22 questions. Among them, 23% (5/22) of topics were related to the PCOS definition, 14% (3/22) to availability of a cure, 14% (3/22) to achieving pregnancy, 14% (3/22) to diagnosis and testing, 9% (2/22) to weight control, 9% (2/22) to metformin, and 5% (1/22) related to other issues.

**Top 10 online questions related to polycystic ovary syndrome.**

What is polycystic ovary syndrome (PCOS)?

How to get pregnant with PCOS?

How to lose weight with PCOS?

What causes PCOS?

How to treat PCOS?

How is PCOS diagnosed?

What does metformin do for PCOS?

Can PCOS be cured?

Can PCOS go away?

What is PCOS symptoms?

### Obstetrics and Gynecology Residency Training

The APGO website provided the proportion of REI rotations among Ob/Gyn residencies in the United States. Of the 206 listed Ob/Gyn programs, six were excluded because of the lack of information about rotations. Of the remaining 200 Ob/Gyn programs, 57.5% (115/200) were university-based programs, and 42.5% (85/200) were non–university-based programs. University-based Ob/Gyn programs had 722 fourth-year residents listed, whereas non–university-based Ob/Gyn programs had 376 such residents. On average, university-based and non–university-based Ob/Gyn programs spent 4% (2/41) and 4% (2/46) of their residency blocks in REI, respectively. A formal REI rotation (ie, at least one complete block) was part of the Ob/Gyn residency curriculum in 92.0% (184/200) of programs, whereas 5.5% (11/200) did not list any REI rotation among their blocks.

## Discussion

### Principal Findings

This study demonstrates that the number of PCOS-related searches by Google has progressively increased over the past decade, with a heightened interest in PCOS shared by 98.2% (716/729) of respondents in our survey. The most common PCOS-related questions searched by Google pertained to its causes, definition, management, and natural history, with the most common PCOS-related question searched by Google being “What is PCOS?”. Ironically, this question is not addressed by the American College of Obstetricians and Gynecologists patient education document about frequently asked questions for PCOS [21].

Specifically, 57.3% (435/759) of respondents in our survey were dissatisfied with their health care, with 42.0% (319/759) and 51.2% (389/759) of the same individuals being dissatisfied with explanations regarding the cause or treatment of PCOS, respectively. Only one in 5 of our respondents had joined an online PCOS support group or forum perhaps because of their preference for independent learning or anxiety to share personal experiences with others [22]. Furthermore, 42.4% (322/759) of survey respondents sought care from a health care provider other than the one who diagnosed PCOS.

A recent survey of gynecologists and reproductive endocrinologists by Dokras et al [2] has shown that 27.2% of these clinicians do not know the diagnostic criteria for PCOS they used. Although more than 85% of clinicians were aware of cardiometabolic comorbidities, fewer gynecologists recognized in patients with PCOS the possibility of concomitant mood-affective disorders, reduced quality of life, or the benefits of lifestyle modification [2].

These findings raise concerns regarding Ob/Gyn resident education on the diagnosis and management of PCOS-related reproductive and metabolic abnormalities, particularly as obstetrician/gynecologists were responsible for diagnosing PCOS in 60.9% (462/759) of our survey respondents. Our data further show that Ob/Gyn residency programs in the United States provide on average only 4% (2/43) of total block time to REI, with 5.5% (11/200) of such residencies not offering any REI rotation at all. Although Ob/Gyn residency programs often address the management of common PCOS-related symptoms, such as irregular menses and excess hair growth, such programs likely limit the clinical exposure of residents to complex PCOS-related metabolic and reproductive abnormalities that often exist within the context of assisted reproduction. Improved Ob/Gyn resident education on PCOS requires a revised curriculum that carefully integrates REI with primary health care aspects of Ob/Gyn. In this manner, REI specialists can interact with Ob/Gyn generalists to provide residents with a complete understanding of PCOS, along with its adverse reproductive and metabolic consequences and individualized clinical management.

Obstetrician/gynecologists also need to maintain their knowledge of recent advances in the field of PCOS. In July 2018, a new set of international guidelines was published for diagnosing and treating PCOS [23] with the goal of improving the clinical care of women with PCOS by physicians of various specialties. Continuing medical education courses that address up-to-date clinical guidelines could aid in this goal, particularly if they incorporate interactive cases or modules aimed at improving patient outcomes [24]. In support of this goal, professional societies should continue to promote interactive physician education, whereas government funding for PCOS research should be increased from its currently underfunded state (vs other chronic conditions) [25] to provide personalized, state-of-the-art health care for all women with PCOS based on multidisciplinary translational research.

The strengths of our study include the innovative use of Google to assess public internet searching behaviors regarding PCOS vs another highly prevalent gynecologic disorder. Information acquired over a decade regarding public interest in PCOS and questions asked online, supported by our survey, call for Ob/Gyn residency programs to improve clinical training in PCOS and for professional societies to maintain relevant educational materials.

### Limitations

Limitations to our study include uncertainty as to whether individuals searching the internet for PCOS information were women affected by this syndrome or not. Sampling bias toward individuals dissatisfied with PCOS health care may also have affected our survey. In addition, information was unavailable regarding PCOS-related questions answered correctly by the CREOG examination as a measure of residents’ competency in PCOS. In addition, the APGO and American Medical Association Fellowship and Residency Electronic Interactive Database websites may not have accurately represented the present Ob/Gyn residency curricula.

### Comparison With Prior Work

Our findings agree with those of previous studies, in which women with PCOS seek health information on the internet [7] in a manner similar to that of individuals with other health issues [26]. That internet searches regarding PCOS exceeded those of fibroids, a highly prevalent gynecologic disorder, supports a previous report of significant patient dissatisfaction with health care regarding PCOS [4]. Unfortunately, sources such as teenagers’ and other women’s digital magazines have social values and beliefs about women with PCOS embedded in the articles [27], further highlighting the need to provide patients with accurate and satisfactory information at the time of diagnosis.

In addition, 42.4% (322/759) of survey respondents sought care from a health care provider other than the one who diagnosed PCOS. In support of this, 60% of women in a previous study sought more than one health care provider before being diagnosed with PCOS [4,5].

### Conclusions

Growing public use of internet PCOS search items above that of other highly prevalent gynecological disorder accompanies growing patient dissatisfaction with PCOS-related health care. Improved clinical care for women with PCOS, combined with continued scientific advances in this important area of women’s health care, calls for academic medical institutions to improve education for clinicians to maintain current knowledge and for government agencies to increase research funding for PCOS as the most common endocrine disorder in women.

## Abbreviations

APGO: Association of Professors of Gynecology and Obstetrics
CREOG: Council on Resident Education in Obstetrics and Gynecology
NIH: National Institutes of Health
Ob/Gyn: obstetrics and gynecology
PCOS: polycystic ovary syndrome
REI: reproductive endocrinology and infertility
SEO: search engine optimization
UCLA: University of California, Los Angeles
